# Concomitant diagnosis of *Trypanosoma cruzi* and HIV in a patient with neurologic manifestations: A case report

**DOI:** 10.1016/j.bjid.2024.104478

**Published:** 2024-11-15

**Authors:** Emmanuel Molina Solano, Mauricio Mora Díaz, Stephanie Montoya Madriz, Mauricio Lizano Calvo, Lissette Retana Moreira

**Affiliations:** a Laboratorio Clínico, Hospital Dr. Maximiliano Peralta, Caja Costarricense de Seguro Social (CCSS), Cartago, Costa Rica; b Departamento de Infectología, Hospital Dr. Maximiliano Peralta, Caja Costarricense de Seguro Social (CCSS), Cartago, Costa Rica; c Departamento de Parasitología, Facultad de Microbiología, Universidad de Costa Rica, San José, Costa Rica; d Centro de Investigación en Enfermedades Tropicales (CIET), Universidad de Costa Rica, San José, Costa Rica

**Keywords:** *Trypanosoma cruzi*, HIV, neurologic, nifurtimox

## Abstract

American trypanosomiasis or Chagas disease is a vector-borne infection caused by the protozoan parasite *Trypanosoma cruzi,* characterized by acute and chronic phases; reactivations due to immunosuppression can occur. In this case report, we confirm the presence of trypomastigotes of *T. cruzi* in a patient with neurologic manifestations. For this purpose, a battery of techniques, including direct examination of cerebrospinal fluid, Giemsa stains, sample cultures, serology and molecular techniques were employed. The patient was treated with nifurtimox for 6 months and started antiretroviral therapy as the concomitant diagnosis of HIV was also performed, showing no sequelae nor adverse effects. A follow-up of the patient´s health status was performed for 42 months.

## Introduction

American trypanosomiasis, also known as Chagas disease, is a vector-borne anthropozoonosis, endemic from the southern region of the USA to the northern part of Argentina and Chile, comprising 21 countries.^1^ The disease is caused by the protozoan parasite *Trypanosoma cruzi* (*T. cruzi*), transmitted by blood-sucking bugs (also known as “kissing bugs”) after the contact with faeces that contain the infective metacyclic trypomastigotes. However, other routes of transmission include blood transfusion, vertical transmission, organ transplantation and the consumption of *T. cruzi* contaminated food and/or drinks.^2^ Nowadays, the current scenario of the disease is changing to also affecting non-endemic countries, turning the disease into a worldwide public health concern.^1^

The pathology of Chagas disease is complex, heterogeneous and dependent on many variables, including parasite and host determinants of immunity and inflammation, which have critical roles.^3^ In this disease, two clinical forms are recognized: acute and chronic. After the primary infection, the acute phase is, in most cases, asymptomatic, but characterized by a high-grade parasitemia;^1^ however, symptomatic manifestations including prolonged fever, headache, myalgia, lymphadenitis, hepatomegaly and splenomegaly can be found.^1^ If vectorial transmission occurred, the infected individual could present clinical signs resulting from the inoculation of *T. cruzi* at the portal of entry: chagoma (entry through the skin) or Romaña's sign (entry through the periorbital mucosa).^4^ After 4 to 8 weeks, the parasitemia decreases, and the clinical manifestations disappear in ∼90% of the cases, when the disease enters the indeterminate chronic phase.^5^ During this phase, the infection remains clinically silent for life in 60% to 70% of cases, but cardiac, digestive (megacolon and megaesophagus syndromes), cardio-digestive or neurological (rare) manifestations can occur in 30% to 40% of cases; of these, cardiac involvement is the most serious manifestation of the disease and affects 1/3 of infected individuals at some point in their lives.^6^ Chronic Chagas disease is considered a disabling disease, responsible for the most significant morbidity and mortality among parasitic diseases.^3^

The treatment of Chagas disease includes benznidazole and nifurtimox as antiparasitic drugs, but these are indicated for patients with acute infection, in congenital cases, in children and recently infected persons, and in reactivation due to immunosuppression.^7^ However, to perform the diagnosis during the acute phase is usually rare, since acute symptoms are easily confused with other infections and illnesses and, when diagnosed and treated, treatment is often discontinued due to a required prolonged course and various adverse effects.^1^^,^^8^ For the chronic phase, there is no consensus for treatment since cure rates are low. It is important to emphasize that the cure rate and its confirmation depend on factors such as phase and duration of disease, age of the patient, tests employed for the evaluation of therapeutic efficacy, time of follow-up after treatment, associated comorbidities and even susceptibility of the *T. cruzi* genotype to the anti-parasitic drugs employed.^9^

In Latin America, it is estimated that 6 to 7 million people are infected with *T. cruzi* and, in endemic countries like Argentina, Brazil and Bolivia, 1% to 32% of HIV infected persons are co-infected with *T. cruzi*.^10^ The infection with Human Immunodeficiency Virus (HIV) causes an immune suppression and CD4+ T-cell depletion occurs, as the virus affects this type of cells. In this sense, some authors consider that coinfections of this virus with other infectious agents can increase the possibilities of progression to AIDS, as the viral replication depends on cellular activity induced by the presence of these pathogens due to the ability to activate Toll-like receptors, and, in an indirect way, by the secretion of pro-inflammatory cytokines or T-cell responses related to the innate immune response.^11^, ^12^, ^13^ Moreover, for Chagas disease reactivation cases in HIV-infected patients with neurological manifestations, there is a debate regarding the moment in which anti-retroviral therapy should be started once the antiparasitic treatment has begun; a major concern, particularly in HIV-infected patients with Central Nervous System (CNS) lesions on HAART, is the potential development of Immune Reconstitution Inflammatory Syndrome (IRIS), which can cause complications.^13^ In cases of meningoencephalitis due to *T. cruzi,* treatment with benznidazole (5 mg/kg) is recommended (doses are divided in two and administered for 60 to 90 days). Some authors also suggest a secondary period of prophylaxis with benznidazole (3 times per week). On the other hand, the use of nifurtimox is an alternative treatment, but clinical experience is limited.^13^^,^^14^

### Case report

A fifty-year old male without known pathologic background consulted the emergency unit of a hospital in Costa Rica due to nausea, headaches and a progressive functional impairment, becoming a dependent person for basic activities of daily living. The patient reported symptoms started after a cranioencephalic trauma that occurred approximately 3 months before attending the health center.

**Fig. 1:**
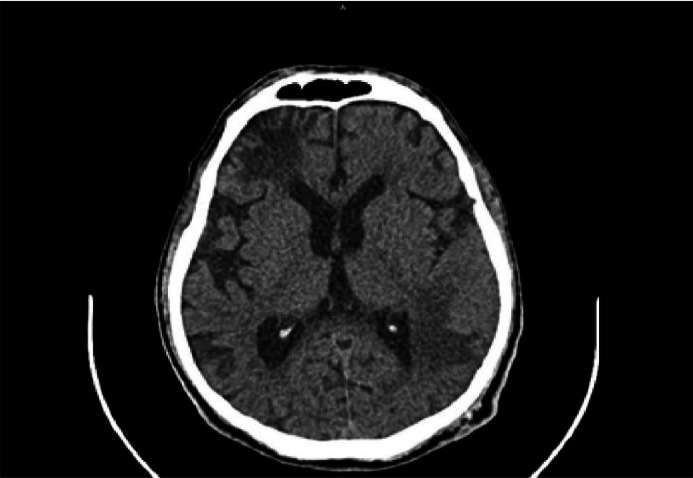
Brain computed axial tomography imaging of the patient that shows left frontal subcortical hypodensity, a left temporo-parietal hypodense lesion.

At the time the patient attended the emergency unit he was oriented but presented a compromise of executive functions like calculation and planning, also showing right patellar hyperreflexia. Computed Tomography (CT) of the central nervous system with contrast medium demonstrated left frontal subcortical hypodensity and left temporo-parietal hypodense lesion (Fig. 1); both lesions did not capture contrast medium. CT also showed extensive subcortical atrophy, without displacement of lesions in the midline. No other lesions were identified.

**Fig. 2:**
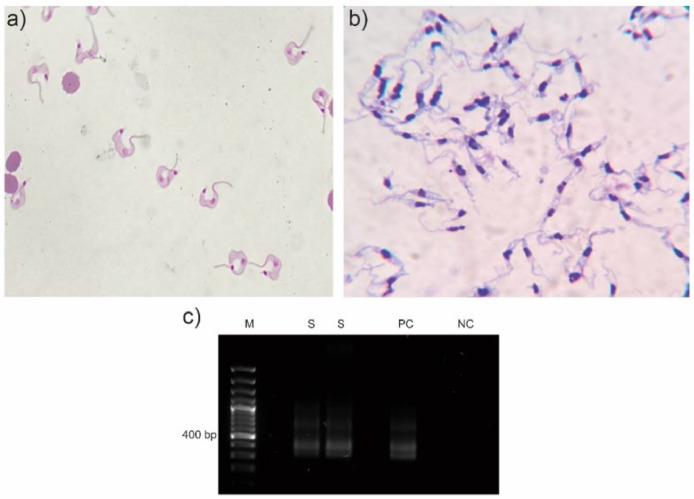
Diagnosis of *Trypanosoma cruzi*: (a) Giemsa stain of the CSF sample that revealed the presence of trypomastigotes; (b) Giemsa stain of epimastigotes after the culture procedures applied; (c) PCR that confirms the diagnosis of *T. cruzi*. M, Gene ruler 100 bp DNA ladder (Thermo Fisher, USA); S, sample; PC, positive control (DNA from *T. cruzi* TCR4); NC, negative control.

A cerebrospinal fluid sample (CSF) of the patient was collected and sent to the clinical laboratory of the hospital for biochemical, bacteriological and parasitological analyses, revealing a decreased concentration of glucose and an increased concentration of microproteins (Table 1). The sample was centrifuged at 2500 rpm for 5 min and the sediment was placed into a Neubauer chamber for leucocyte and erythrocyte count. During the visualization under the microscope, the presence of high-motile trypomastigotes was observed (Fig. 2). This sediment was fixed using methanol and Giemsa stains were performed.

**Table 1:** Biochemical analysis of the patient's CSF sample.

Analysis	Value
Volume	3.0 mL
Glucose	30.0 mg/dL (reference: 50.0 – 80.0 mg/dL)
Microproteins	69.0 mg/dL (reference: 14.0 – 45.0 mg/dL)
Lactate dehydrogenase	41 UI/L
Leucocytes	3 mm^3^
Erythrocyte	350 mm^3^

The same day, after the identification of motile trypomastigotes in the sample, a serum sample was also collected for IgG detection using the ARCHITECT Chagas chemiluminescence assay (Abbott, USA), evidencing the presence of IgGs anti *T. cruzi* with paramagnetic microparticles coated with recombinant antigens (FP3, FP6, FP10 and TcF).

An aliquot of the CSF sample was transported to the Faculty of Microbiology of the University of Costa Rica for culture in Vero cells and then, in LIT culture medium for epimastigote growth and parasite´s isolation. DNA extractions and PCR analyses using the pair of primers 121 and 122, which target to the 330-bp variable regions of the *T. cruzi* Kinetoplastid Minicircle genome (kDNA)^15^ were also performed (Fig. 1). Another aliquot was sent to a reference center (INCIENSA), which also confirmed the presence of *T. cruzi* using molecular and serological techniques.

The patient was treated with nifurtimox (240 mg every 8 hours) for 6 months after the diagnosis was performed at the health center.

Concomitantly, an HIV infection was diagnosed, evidencing a CD4+ T-lymphocyte count of 69 cells/mL (CD4/CD8 ratio: 4%) and a plasma viral load of 2180,000 copies/mL (6.34 log). Other opportunistic infections were ruled out, including the infection with *Toxoplasma gondii* by RT-PCR (not detected). The presence of *Cryptococcus neoformans* was also discarded after Indian ink stain and RT-PCR. Negative syphilis studies were documented in both serum and CSF samples. *Mycobacterium tuberculosis* was ruled out after PCR of CSF and negative CSF cultures for Mycobacteria.

Antiretroviral treatment (ART; TDF/FTC/EFV) was initiated 17 days after starting nifurtimox. Recovery of neurological symptoms was documented after 2 weeks of treatment with nifurtimox, without secondary effects reported by the patient. Moreover, no evidence of cardiovascular compromise was found, showing preserved biventricular systolic function by echocardiogram.

The patient was discharged a week after starting the antiretroviral therapy, with follow-up appointments at the infectious disease unit of the hospital. A recovery of higher mental functions was also documented, achieving independence in activities of daily living. The patient also presented immunological response, achieving viral suppression 3 months after starting ART. Recovery of immune function was also achieved, with improvement in CD4+ T-lymphocyte count and undetectable HIV viral load (Table 2).

**Table 2:** CD4+ T-lymphocyte count and quantification of HIV viral load of the patient: follow up during 42 months.

Time point	CD4+ T-Lymphocyte count	HIV viral load (copies/mL)
Initial diagnosis	69	2,180,000
2-months	271	685
6-months	391	28
18-months	431	Undetectable
30-months	489	31
42-months	Not performed	Undetectable

Nowadays, the patient does not present persistent neurological damage, has complete viral suppression and recovery of the immune function. The patient keeps assisting to his control appointments in the infectiology unit of the health center.

## Discussion

A report of reactivation of Chagas disease with neurological involvement in an HIV-coinfected patient is performed in this study. Neurological involvement of this disease can be defined as the finding of 1) trypomastigotes in the direct examination of cerebrospinal fluid, 2) amastigotes in the histopathological analysis of brain tissue, or 3) trypomastigotes in the direct examination of blood, associated with neurological manifestations and clinical response after specific treatment.^16^ In this case, trypomastigotes were observed in a CSF sample and several techniques were applied to confirm the diagnosis and then, to isolate the parasite.

Neurological compromise is considered the most common presentation in cases of reactivation of Chagas disease.^17^ In these cases, the involvement of the central and the peripheral nervous system is not frequent, but may result in dementia, confusion, and neuritis.^18^ Usually, reactivation of Chagas disease in chronically infected patients may result in neurological findings secondary to the presence of intracerebral mass lesions or acute diffuse meningoencephalitis. On the other hand, it has been reported that only a small number of patients infected with *Trypanosoma cruzi* develop encephalitis during the acute phase, the majority of whom are 2 years old or younger and present encephalitis invariably associated with myocarditis.^18^

Chagas disease reactivation is usually associated with immunodeficiency disorders, such as hematological malignancies, solid organ transplantations or AIDS, and exhibits a high mortality rate in AIDS patients.^17^ It presents with neurological compromise in 75%‒90% of patients, and acute myocarditis in 30% of patients;[17] other uncommon presentations include peritonitis, pleural effusion, cervicitis and skin lesions.^19^ Neurological manifestations of Chagas disease reactivation depend primarily on the location, size, and number of lesions, which can vary.^18^

In 2008, Cordova et al. published one of the largest reported series of HIV-infected patients with neurological involvement due to Chagas disease.^16^ In their study, the authors reported headache, focal neurological deficits and fever as the most common manifestations of CNS involvement of the patients, which are indistinguishable from other causes of meningoencephalitis. Moreover, 13 from 15 patients presented abnormal neuroimages, being the most frequent finding a single supratentorial hypodense lesion compatible with abscess, predominantly involving the white matter of the brain,^16^ findings that were similar to the ones reported in literature, according to the authors.

In a review of 100 years of neurological involvement of Chagas disease,^19^ all the 180 patients described had underlying immunosuppression. According to the author, all patients with AIDS and reactivation of the disease in the CNS had CD4+ T-lymphocyte count of less than 200 cells, with a high mortality rate. However, there was at least one report of a patient with meningoencephalitis not related to immunosuppression. Fernandes et al. also reported another case in which reactivation with CNS involvement occurred in a patient without overt underlying immunodeficiency.^18^

As previously described, CNS compromise is usually confirmed by the microscopic identification of trypomastigotes in CSF, histopathological analysis of brain biopsies or positive Strout method result.^17^ As there is limited information regarding the use of molecular techniques like PCR conclusively in samples such as CSF and tissues,^17^ parasite cultures were established and PCR was performed using epimastigotes grown in LIT culture medium, a more time-consuming approach but allowed the isolation and maintenance of the parasite in laboratory conditions; however, molecular diagnosis by PCR was also confirmed by INCIENSA using CSF. It is important to highlight that, although several techniques were employed for a complete diagnosis of Chagas disease in this patient, rapid treatment was established since trypomastigotes were identified in the CSF sample and IgGs anti-*T. cruzi* were detected by chemiluminescence. In this case, nifurtimox was administered and the patient recovered, with no signs of neurological damage.

As suggested by Kaushal et al.^20^ we support the proposal that Chagas disease should be included in the differential diagnosis when an immunocompromised patient presents with CNS masses, meningoencephalitis or focal neurologic signs, as well as the fact that successful treatment of Chagas disease requires a high index of suspicion for timely diagnosis.

## Ethic's statement

The publication of this case report was approved by the Bioethics Committee of Centro de Desarrollo Estratégico e información en Salud y Seguridad Social (CENDEISSS), of Caja Costarricense de Seguro Social (CENDEISSS-AB-0319–2024).

## Funding

This research did not receive any specific grant from funding agencies in the public, commercial, or not-for-profit sectors.

## Conflicts of interest

The authors declare no conflicts of interest.
